# Beyond Diffusion: Harnessing Physical Principles for Precision KRT

**DOI:** 10.2215/CJN.0000000917

**Published:** 2025-10-01

**Authors:** Franklin W. Maddux, Ryan A. Jimenez

**Affiliations:** Fresenius Medical Care AG, Bad Homburg, Germany

**Keywords:** dialysis, ESKD, KRT

CKD is a major global public health crisis, affecting over 800 million individuals worldwide with over one million deaths annually from untreated kidney failure. CKD is now a global health priority with the World Health Organization's landmark May 2025 resolution to reduce noncommunicable disease burden by promoting kidney health and strengthening kidney disease prevention and control.

Despite significant advances in dialysis and transplantation over the half century that these KRTs have been available, kidney disease burden remains substantial in terms of mortality and economic impact. In 2022 alone, costs in the United States associated with CKD were estimated at $48 billion. The growing recognition that conventional KRT often fails to address the complexity and individuality of a person's needs compounds these challenges.^1^

Historically, KRTs became reliable methods with standardized treatment protocols, offering life-sustaining care but limited personalization. As CKD understanding deepens—particularly the interconnectedness with cardiovascular disease, hypertension, diabetes, obesity, and other comorbidities—the need for more tailored, precise approaches become increasingly desirable.

Innovations rooted in fundamental physical principles like diffusion, convection, adsorption, and gas exchange offer a promising roadmap to reimagine KRT. This perspective explores how applying these physical principles to personalize KRT can lead to significant improvements in survival, quality of life, and treatment burden, addressing unmet patient needs while supporting sustainable health delivery. By leveraging advances in diagnostics, dynamic therapy adjustment, and integrated patient monitoring, nephrology stands at the threshold of an era of opportunity—one where therapy is both standardized yet individualized.

CKD is a progressive, multifactorial condition with profound public health implications. It often develops silently and is underrecognized until advanced and closely tied to comorbid chronic conditions. Beyond clinical burden, CKD imposes significant societal and economic costs, exacerbated by health care access inequities and disparities in disease prevalence globally.^1,2^

Although life-prolonging, current KRT options—including hemodialysis, peritoneal dialysis, and transplantation—frequently fall short of restoring optimal health. Patients often experience high hospitalization rates, reduced functional capacity, cognitive decline, cardiovascular complications, and impaired quality of life. Moreover, therapies have traditionally followed a one-size-fits-most model, with minimal patient individualization.^1–3^

Addressing these challenges requires a novel vision for KRT—one that recognizes each patient's unique physiological profile where therapy is no longer static but dynamically tailored to a patient's evolving health status. This approach requires the full nephrology community including academia, industry, and payers to enhance diagnostic precision which informs therapy choices, incorporating advanced diagnostics, and individualized care plans.^3^

Patient engagement is a central feature: Empowered patients actively participating in managing their therapy have better outcomes. Advanced technologies, including genomic testing, continuous physiological monitoring, and artificial intelligence (AI), are key enablers, providing real-time insights to fine-tune more personalized treatment strategies. Personalization extends beyond dialysate and fluid balance adjustments; it demands a holistic appreciation of comorbidities, functional capacity, cognitive function, and cardiovascular health, all integrated into a comprehensive, adaptable care platform.

At the heart of innovative KRT lies a set of fundamental physical principles that can be used to modify human blood composition:^4^

○ Diffusion involves the passive movement of solutes from an area of higher concentration to lower concentration across a semipermeable membrane. It remains the cornerstone of traditional hemodialysis for removing waste products like urea and creatinine.^4,5^
○ Convection moves solutes alongside water across membranes, driven by transmembrane pressure gradients. Hemodiafiltration, combining diffusion *and* convection, enhances the removal of middle molecular weight toxins while offering an exchange of plasma water.^6^
○ Adsorption selectively binds specific molecules to a membrane surface, offering a mechanism to remove inflammatory mediators or specific molecules.^5,6^
○ Gas exchange ensures the adequate transfer of oxygen and carbon dioxide, preventing hypoxia and associated organ dysfunction, an often underappreciated aspect of KRT management.^7–9^

**Figure 1:**
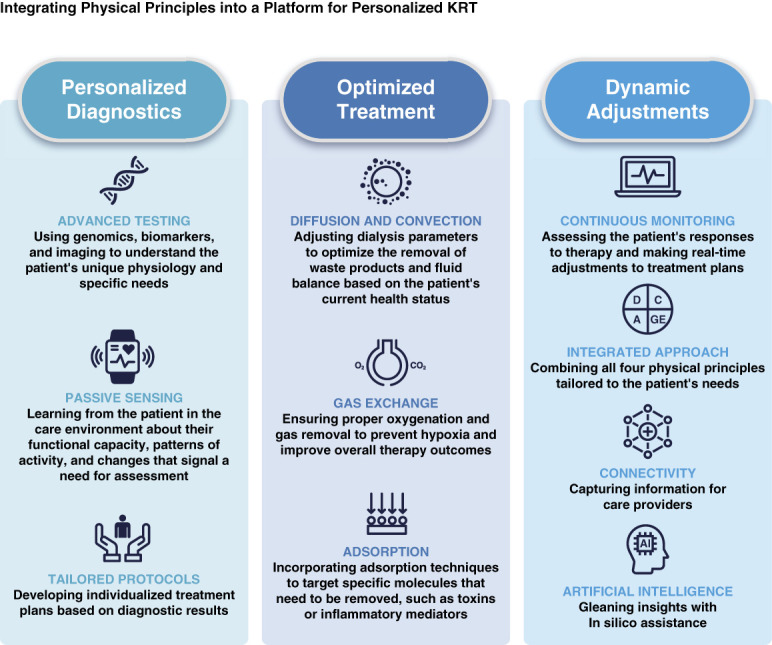
Personalized KRT functional map.

Harnessing these principles optimizes solute clearance and supports broader physiological stability, opening avenues for more effective, personalized therapies (Figure 1).^8–10^

Diffusion's limitations alone prompted innovations such as hemodiafiltration, which additionally incorporates convection. This pairing of two physical principles provides a roadmap of how other blood modification methods might further enhance therapy.^10^

Recent studies, including the landmark CONVINCE trial, demonstrated that hemodiafiltration offers superior quantity and quality-of-life outcomes for patients undergoing dialysis compared with conventional high flux hemodialysis. Meta-analyses and real-world evidence encompassing tens of thousands of patients further suggest that high-volume hemodiafiltration is associated with improved survival, reduced cardiovascular mortality, and lower treatment burden. Economic evaluations support the broader adoption of these approaches, highlighting cost-effectiveness when considering long-term benefits of reduced morbidity and hospitalization.

Although diffusion and convection enhance solute clearance, there remains to be addressed the complex inflammatory milieu in dialysis patients. Adsorptive technologies aim to remove cytokines, endotoxins, and other pathogenic molecules, offering targeted means to modulate systemic inflammation. Furthermore, evidence of specific pathogen and protein bound uremic toxin removal offer the chance to tailor therapy to reduce an individual's comorbid burden such as the neurocognitive decline from prolonged ESKD.

In parallel, gas exchange addresses the known impact of prolonged intradialytic hypoxemia which leads to progressive cardiovascular risk and dysfunction. Such risks from inflammation and alveolar capillary blockade in the lungs can be mitigated through extracorporeal oxygenation during KRT.

Personalized KRT necessitates a diagnostic infrastructure capable of deep patient profiling. Genomic and biomarker analysis, imaging technologies, and environmental sensing combine to provide data-driven insights and treatment decisions.

Research programs like those from the startup Nephronomics represent a significant step forward, building a large-scale whole-genome sequencing and clinical data repository on over 35,000 people with advanced kidney disease. Insights from genetic study reveal that many cases of CKD previously labeled under legacy diagnostic categories may, in fact, result from identifiable, sometimes targetable, gene mutations, expression, and variants. Both primary variants and epigenetic modifiers of gene expression will enable more precisely diagnosed conditions and aid specific treatment regimens through pharmacogenomic insights.

Continuous monitoring devices, connected care platforms, and AI-driven analytics enable real-time therapy adjustments, ensuring treatments evolve with the patient's changing condition, enhancing chances for better clinical outcomes and a safer treatment environment.

Looking ahead, the vision for KRT expands what care looks like and is transformative in treating gaps that exist today in comorbidity management. Future strategies will:

- Allow nephrologists to flexibly personalize therapy across a broad spectrum of patient phenotypes and conditions.
- Target neurocognitive dysfunction, cardiovascular risk, and oxygenation deficiencies that occur with KRT.
- Leverage regenerative medicine approaches, including cell-based therapies, tissue engineering, and genetic modifications.
- Use AI for predictive modeling, treatment optimization, and safety surveillance.
- Recognize and adapt therapies to genetic signatures influencing individual drug response and disease progression.
- Given the substantive environmental burden of dialysis, the nephrology community holds a collective responsibility to advance a future of more sustainable therapies.

By the next decade, future KRT has the opportunity to offer deeply personalized interventions that restore many aspects of not only kidney function but overall health and vitality. Applying the physical principles of diffusion, convection, adsorption, and gas exchange to personalize KRT holds the promise to fundamentally change patient outcomes. By integrating cutting-edge diagnostics, continuous monitoring, and AI-driven insights, nephrology can move from standardized treatments to truly individualized care. The future of KRT is not only about sustaining life, but enhancing it—through scientific precision, technological innovation, and compassionate personalization.
